# REPEAT TRIAGE IN DISASTER RELIEF: QUESTIONS FROM HAITI

**DOI:** 10.1371/4fbbdec6279ec

**Published:** 2012-10-22

**Authors:** Nir Eyal, Paul Firth, MGH Disaster Relief Ethics Group, L Black, AFM Cist, M Curran, AN Dalal, AK Goodman, A Le Roy, N Sunder, MC Wilson

**Affiliations:** Harvard Medical School; Harvard Medical School and Massachussetts General Hospital

## Abstract

During a mass casualty disaster, the acute imbalance between need for treatment and capacity to supply care poses difficult rationing problems. It is common to assume that such disasters call for “utilitarian” procedures that deliberately prioritize saving the most lives over other considerations. A group of medical responders to the 2010 Haitian earthquake faced particular challenges in determining how to allocate limited treatment, time and other resources between existing patients and potential patients not yet under care. We identified that rationing dilemmas points occurred at three points: when care had to be limited, when care had to be completed prematurely, and when care had to be withdrawn. “Repeat triage” refers to rationing challenges occurring at all these points, where the allocation of care is between existing and potential patients. By contrast, “initial triage” designates the allocation of access to treatment among new arrivals, all of whom are potential patients. Repeat and initial triage differ significantly. Several considerations make repeat triage special by supporting limited priority to existing patients, in transgression of pure “utilitarian” procedures: (1) Pragmatically, often it is more efficient to complete treatment on existing patients, for whom prognosis can be established with greater certainty and without added time, than to attempt to save new patients; (2) A fiduciary trust relationship has been formed between care-giver and existing patients, which may make the moral obligation towards them somewhat stronger than the one to potential patients; (3) Existing patients will have often arrived earlier, so when needs are equal, the “first come, first served” principle prioritizes them for care; (4) Withdrawal of care during repeat triage may constitute active rather than passive harm, and more often a serious transgression of patient autonomy; (5) Health providers should normally not be asked to behave in ways that profoundly violate their personal and professional integrity, and abandoning existing patients may do so. For these reasons, responders can permissibly give a degree of priority to existing patients over newcomers in disaster.

## Introduction

The earthquake that struck Port-au-Prince, Haiti on January 12^th^ 2010, killed and injured hundreds of thousands. With medical needs far exceeding available resources in this massive disaster, medical teams encountered severe ethical challenges. Rationing dilemmas in a mass casualty situation commonly evoke the initial triage of casualties, where whom to treat first is being decided. The triage decisions that posed the most difficult moral quandaries for some medical teams in Haiti, however, arose after the primary triage into treatment. These medical responders wondered when and how they should limit medical care for patients who were already under care with their team, in order to reserve resources for new casualties who were also in need of treatment.

In the first weeks after the earthquake, approximately eighty-five volunteers from the Massachusetts General Hospital (MGH) joined the relief efforts. These responders worked in MGH groups and non-governmental organizations based in small rural Haitian hospitals; with an International Medical and Surgical Response Team; and aboard the USNS Comfort, a US Navy hospital ship that served as the major referral hospital. Upon their return to Boston, a group met to discuss the ethical dilemmas that they had faced.

In order to clarify the differences in rationing method appropriate to different stages of care, we distinguish between primary and subsequent triage points. We use the term “initial triage” to describe the sorting of casualties on arrival into categories of priority for treatment - the primary rationing decision as to whom to treat first. We define “repeat triage” as rationing treatment between patients who had already been accepted into care and those who were not (yet) under care. While “initial triage” allocates care between new arrivals, “repeat triage” allocates care between existing and potential patients.

We identified three “cases”—types of situation in which group members had to consider limiting care for existing patients in order to maintain resources for new arrivals who were not yet within the medical system. We discuss the thought processes that went into clinical decision making before analyzing the ethical considerations that bear the most on these dilemmas, with reference to the relevant ethics literature.

In care rationing, several ethical considerations demarcate existing from potential patients and hence, the decision making appropriate for repeat triage from the one appropriate for initial triage. They include the pragmatic advantages to treating existing patients, the fiduciary relationship that links an existing patient to a care giver, the allocation principle of first-come first-served, the doing-allowing distinction linked to patient autonomy, and the rights and motivation of health care workers.

## Possible Cases

**Case 1: Limitation of care.** A child is anesthetized for washout of an open fracture of the tibia, fibula and foot. On opening the dressing, the foot is found to be infected and open to the bone. A conservative option would be to wash the wound out and treat with antibiotics, in the hope that it heals sufficiently to bring down a muscle flap end to cover the open bone at a later point. This would require hours of operation, combined with anticoagulation and close follow-up to keep the muscle flap from thrombosis. There is a huge backlog of urgent cases requiring life- or limb-saving operations. An operation, even days later, would delay other urgent cases. Operating room staff discusses the case. The difficulty of ensuring prolonged anticoagulation when nursing care is severely limited markedly diminishes the chance of a muscle flap succeeding. Alternatively the child could be airlifted to the United States. A comment is made that there are thousands of cases just like this, and it is impossible to airlift everyone.

**Case 2: Premature completion of care.** The hospital is filled to capacity; new patients cannot be admitted for life- and limb-saving treatment until existing patients are discharged. A newly paraplegic patient has been treated and provided with initial care. The patient is to be transferred to a Haitian facility. But there is very limited ability to care for paraplegics in Haiti, and, if discharged, the patient will almost certainly develop potentially-fatal bedsores or urinary tract infections. Still, the need to discharge existing patients in order to admit new patients is pressing. Strong disagreements occur amongst relief team members, scheduled to leave shortly, as to when it is appropriate to discharge patients. Similar problems with discharge follow-up abound: external fixators have been placed on fractured limbs, but facilities to remove the hardware in several weeks’ time do not exist; a child with kwashiorkor improving with adequate food will relapse if discharged to the inadequate environment from which they came; the infrastructure for months of outpatient treatment of a child with newly diagnosed tuberculosis does not exist.

**Case 3: Withdrawal of care.** A patient develops severe complications while intubated and ventilated for an operation in a small hospitall. Fourteen hours post-operatively the patient is still ventilator-dependent. The cause, outcome and time-course of the underlying disease are uncertain. There are one anesthesia machine and one anesthesiologist, needed for urgent and emergent surgeries on other patients. With great distress, the team considers whether to extubate the patient so as to allow anesthesia for multiple other victims.

## Discussion

The World Health Organization defines a disaster as a state that occurs when normal conditions of existence are disrupted and the level of suffering exceeds the capacity of the hazard-affected community to respond to it.^1^ There has been extensive discussion of initial triage during disasters. Suggested triage protocols are typically based on striving for the best combined medical outcome, most often, maximizing the number of lives saved, a so-called “utilitarian” procedure.^2^^-^ 7 They reflect the widespread assumption that even if utilitarianism is not an appropriate theory or procedure for normal times, it is appropriate for public health emergencies. At this point care is deliberately rationed based only on differences in medical need and potential individual outcomes.

None of the three cases described above falls into the category of initial triage. These “repeat triage” decision points—treatment limitation, premature completion of treatment, and treatment withdrawal—follow the primary decision about whether to initiate care. Individual care is rationed between existing patients and potential patients. Repeat triage can therefore differentiate individuals according not simply to medical necessity and prognosis, but also, potentially, to their status as patients under medical care or alternatively, as people who have not yet entered care.

Initial and repeat triage may look like two instances of the same basic dilemma—how to reconcile the conflicting needs of the individual for maximal care with other people’s similar needs. But are there ethical or practical differences? Might the propriety of “utilitarian” procedures vary between initial triage and repeat triage? In particular, does the existing patient in repeat triage have some priority a potential patients when prognoses are similar and give neither a higher chance of benefit from treatment?

**Pragmatic Considerations**

The simplest reason to prioritize existing patients during disaster is that, other things being equal, it is often more efficient to prioritize existing patients than to discharge them and admit new patients.^6^ Admissions and discharges carry a certain “friction cost”—time and limited resources spent on moving patients around, fully diagnosing a new case, and so forth. Moreover, as a general rule, physicians will be a little surer about the prognosis and future needs of an existing patient than about those of a new admission.^7^ When the former seems to have an acute need, it is often more efficient to reserve resources for her than for a new admission with what appears to be a similar acute need, since outcome can be determined with greater certainty for an existing patient. Some priority for existing patients is often an efficient “rule of thumb” for maximizing the number of lives saved. As such, it tends to fulfill the utilitarian dictum to maximize benefits. It does so without consciously and directly focusing on that dictum, but what that dictum demands is not conscious focus on that dictum.^8^ There may, however, be deeper ethical reasons to prioritize existing patients—reasons that apply even when, in a particular case, the expected utility is similar.

**Honoring Patients’ Trust**

One argument for prioritizing existing patients somewhat is that a special fiduciary relationship has developed between the care-giver and any existing patient. The moral obligation to any existing patient could thus be considered greater than the obligation to an individual with whom a strong relationship has not as yet formed. Ethicists have accounted for the practitioner’s special obligations to existing patients based, for example, on the trust that these vulnerable persons have placed in him or her and to which he or she agreed to be faithful.^9^^-^ 12 Faithfulness to trust may seem like a consideration whose relative force is far greater in normal times than during disaster, when many lives hang in the balance. However, in some ways trust in physicians becomes especially important during disasters, when medical systems are under high pressure and lives depend on their smooth functioning. A system in which everyone is assured that care, once given, will not be withdrawn, saves patients from the need to petition and network for continued care, and from the anxiety and suspicion that may accompany such ongoing lobbying. Both patients and their physicians are freer to focus on medical care. While in disaster situations, patients rarely have an alternative to cooperation with the medical team, seeing doctors as staunch advocates on their behalves can offer dependent patients some dignity, promote medical compliance and cooperation, and improve the functioning of the medical system in a chaotic time.

**Arrival Time**

An existing patient may be thought to command priority over new arrivals simply based on the blind and imperfect fairness of “first come, first served,” a prevalent approach to allocating medical care, which nearly every health system uses to some degree. While sometimes those who arrive first are those who have more resources, it is hard to identify an alternative that would completely displace that principle, especially in chaotic disaster situations. When applied consistently, this relatively impartial principle can also prevent gross discrimination.^13^ When patients’ needs are extremely unequal, ‘first come first served’ is not a reasonable method of allocating scarce resources.^14^ But when the difference between needs is modest or nil, first come, first served substantiates a moderate priority for existing patients.

**Withholding versus Withdrawing Care**

Initial triage during a disaster asks whom to withhold resources from for the sakes of others. Premature completion of care and care withdrawal—cases of repeat triage during a disaster—ask whom to withdraw resources from for the sakes of others. To take resources away from a patient while she is using them only to serve others is hard to justify; much harder to justify than either taking resources away from a consenting patient for her own sake, or withholding resources from a patient for others’ sakes, before she begins using them. If you will, the synergetic combination of two elements that are individually benign—rationing and treatment withdrawal—makes some cases of repeat triage difficult to defend.

Let us look at the two elements individually. Rationing often disadvantages, and in that modest sense harms, nonrecipients of rationed resources. But in a world of scarce resources some rationing remains necessary.^15^^,^ 16 Standard rationing forces disadvantage only by omission—by failure to hand everyone all the scarce resources that might benefit them. Rationing does not actively harm nonrecipients.

Move to treatment withdrawal. In standard care, treatment withdrawal deliberately benefits a consenting patient and that makes it easy to justify. Even consensual withdrawal of life support (say, from a terminal cancer patient in pain) seems roughly as justifiable as consensually withholding life support from such a patient.^17^

Either rationing or treatment withdrawal are clearly defensible on their own. Returning to repeat triage during disaster relief, however, premature completion of care and care withdrawal bring together both rationing and treatment withdrawal. That combination remains hard to justify, for two reasons.

First, when the impetus behind treatment withdrawal is rationing, not the interests of the patient from whom that treatment is taken, then any disadvantage that this patient consequently incurs may count as active harm done to her, insofar as withdrawal is active.^17^ Active harm is far harder to justify than either treatment withdrawal when it involves benefit not harm, or rationing when it involves omission not action, on their own.

A second way in which care withdrawal pursuant to some cases of repeat triage differs from the mere withholding of care pursuant to initial triage surrounds patient authorization. Outside of disaster situations, care termination for a patient’s own sake (say, termination of pneumonia care in an aging patient with end-stage cancer) can only be withheld with the (advance) consent of the patient (or that of the patient surrogate), and when it is safe to presume that, if the patient could give or refuse rational consent, he or she would consent.^18^ The case is very different when the sole rationale for withdrawing (or withholding) care is third parties’ greater medical needs, and the immediate patient does not consent to it. This challenge to the patient’s autonomy is especially hard when care is withdrawn. Our strongest autonomy rights are negative rights against unwanted intervention, including care withdrawal. Autonomy rights to an intervention—for instance, to not being withheld care—are somewhat weaker. To illustrate, the question whether pro-life practitioners must provide early-pregnancy termination services to women who seek them is complex. But there is no question as to whether such practitioners are allowed to force such women to undergo intervention to save their embryos. They are not. Insofar as withdrawing intervention is more active than withholding it, it raises a harder, and sometimes an insurmountable, ethical challenge when patients refuse it—as they will often do when the motivation is rationing and not their own interests and will. This is not to deny that withholding care from a newcomer for an existing patient’s sake reduces autonomy as well. It affects the newcomer’s ability to decide her fate. Still, such transgressions of autonomy rarely alarm bioethicists and practitioners as much.

While legal recourse is scarcely available to disaster victims, at the extreme end, non-consensual withdrawal of treatment might theoretically be interpreted as assault and, when harmful, as a tort, perhaps especially when it involves the removal of tubes from inside a patient’s body.^6^^,^ 19 ^-^ 21 By contrast, there is no suspicion of battery when as-yet unallocated resources, physically separate from the patient’s body, are shifted to others.

**Health Worker Motivation**

Earlier we mentioned that abandoning an existing patient with whom a deep and a compassionate care relationship has (hopefully) been formed tends to be emotionally more taxing on medical teams than refusing new admissions is. Because repeat triage is being conducted by health practitioners, the importance of their typical emotional response may support some priority to existing patients.

It is tempting to dismiss practitioners’ emotional responses as mere emotional responses with little relevance to fair resource allocation—as we would dismiss the emotions of a bigoted practitioner who fears treating HIV-positive patients. Especially during disaster, some may conclude, surely the extreme urgency of rescuing many patients’ lives overrides protecting practitioners’ emotions. On that view, practitioners should simply learn to prioritize public health needs over their own feelings and prejudices.

However, within limits, practitioners’ normal emotional responses do matter—especially during disaster. First, there are simple pragmatic reasons to avoid extreme emotional distress and protect personnel morale, certainly when stemming not from stigma but from commendable compassion that we generally encourage among medical workers. Preventing emotional burnout and preserving team unity may protect nearly everyone’s prospects, including some untreated patients who rely on future care in a functional care team. Taxing demands on relief workers—including being regularly forced to discharge vulnerable people onto the streets, to remove a critically ill patient from life support, or to amputate a child’s limb against their own sense—can result in worker attrition, system failure, and compromised care during disaster.^6^^,^ 22

Though the argument can rely on pragmatic considerations alone, we should bear in mind that care-givers also have rights, and usually we do not insist that they provide care when that would violate their personal and professional integrity.^23^^,^ 24 For many reasonable physicians, abandoning an existing patient stands in such fundamental contrast to how they were trained to treat patients that it would violate that integrity. Since within limits the health worker has rights to behave in ways that accord with his own basic values, one could make the case that physicians should be granted a moderate prerogative to prioritize existing patients over newcomers. By contrast, when patients present during initial triage, it is clearly unreasonable for medical teams to refuse to admit those patients whose needs are greatest.

Thus, short of transferring more decisions to computers, mechanized decision algorithms, or independent teams who have no part in these patients’ care 3 (which might complicate logistics and waste caretakers’ special knowledge of their own patients), these caretakers’ emotional needs must be considered–which may grant their patients limited priority.

## Practical Implications and Future Directions

Our experiences and deliberation suggest not only that many triage points fall beyond the primary allocation of treatment, but that initial and repeat triage differ in the ethical challenges that they pose. In repeat triage, a moderate priority to existing patients over new admissions is often acceptable.

As planned responses to disaster become more frequent, the expanding academic study of disaster response is providing insights into disaster epidemiology that may guide response patterns. Planners and coordinators of disaster relief efforts should recognize the multiple triage points they involved. Such recognition may allow prior preparation of the infrastructure and guidelines for transfer, discharge and follow-up that check repeat triage challenges.^22^^,^ 25

During relief work, the formation of ethics committees and discussion support groups may further help individuals consider all options, improve decision making, decrease psychological tensions, and diffuse the responsibility for choice with profound consequences from the individual practitioner to the group.^26^^-^ 31 The norms governing such ethics committees and group discussions should allow a moderate priority for existing patients during repeat triage.

We do not advocate an about-face in disaster relief practices, but a modification of the ethos. This moderate priority may turn out to be more closely aligned with what many relief workers already practice. If this analysis is correct, future commentaries should characterize in greater detail the appropriate degree of priority for existing patients.

Other commentaries could examine further questions. Does repeat triage in which both candidates for medical resources are existing patients differ from initial triage (that is, from triage between two new admissions)? Is repeat triage in which the existing patient was admitted before the disaster took place, with the expectation of normal fiduciary relations,^25^^,^ 32 any different? Is the same level of priority to existing patients in disaster settings also appropriate in regular ICUs that perform repeat triage far from disaster zones?^14^

## Conclusion

During disaster relief, a moderate priority to existing patients over new admissions is often acceptable. While absolute or very high priority is unwarranted, pragmatic considerations; the need to respect and preserve the fiduciary trust relationship; the first come, first served principle; distinctions between withholding and withdrawing treatment and their confluence with the notions of active and passive harm and patient autonomy; and the rights and morale of health care providers support a moderate priority for existing patients in repeat triage. Some of these considerations, such as the need to prevent responder team attrition by prioritizing existing patients, are especially acute during disaster—precisely because life and death hang in the balance for many. By inviting deliberation on factors beyond maximizing medical benefits and the number of lives saved, our argument questions the propriety of shifting fully to so-called utilitarian procedures.

## Competing Interests

The authors have declared that no competing interests exist.

## References

[1] World Health Organization. Emergency and Humanitarian Action. 2005.

[2] Christian MD, Hawryluck L, Wax RS, et al. Development of a triage protocol for critical care during an influenza pandemic. CMAJ 2006;175:1377-81. 10.1503/cmaj.060911 PMC1635763 17116904

[3] Hick JL, Rubinson L, O'Laughlin DT, Farmer JC. Clinical review: allocating ventilators during large-scale disasters--problems, planning, and process. Crit Care 2007;11:217. 10.1186/cc5929 PMC2206420 17601354

[4] World Medical Association. WMA statement on medical ethics in the event of disasters. Adopted by the 46th WMA General assembly, Stockholdm, Sweden, September 1994; revised by the 57th WMA general assembly, Pilansberg, ZA, October 2006.

[5] Levin D, Cadigan RO, Biddinger PD, Condon S, Koh HK, Joint Massachusetts Department of Public Health-Harvard Altered Standards of Care Working Group. Altered standards of care during an influenza pandemic: identifying ethical, legal, and practical principles to guide decision making. Disaster Med Public Health Prep 2009;3 Suppl 2:S132-40. 10.1097/DMP.0b013e3181ac3dd2 PMC10353013 19755912

[6] Eastman N, Philips B, Rhodes A. Triaging for adult critical care in the event of overwhelming need. Intensive Care Med 2010;36:1076-82. 10.1007/s00134-010-1862-0 20349037

[7] Yitzhak A, Sagi R, Bader T, et al. Pediatric ventilation in a disaster: clinical and ethical decision making. Crit Care Med 2012;40:603-7. 10.1097/CCM.0b013e318232e222 22020234

[8] Hare R. Rights, Utility and Universalization: Reply to J. L. Mackey. In: Frey R, ed. Utility and Rights. Minneapolis: University of Minnesota Press, 1984:106-120.

[9] Joffe S, Truog RD. Consent to medical care: the importance of the fiduciary context. In: Miller FG, Wertheimer A, eds. The ethics of consent. 1st ed. New York: Oxford University Press, 2010:347-374.

[10] Richardson HS. Incidental findings and ancillary-care obligations. J Law Med Ethics 2008;36:256,70, 211. 10.1111/j.1748-720X.2008.00268.x PMC2575243 18547193

[11] Zaner RM. The Phenomenon of Trust and the Patient-Physician Relationship. In: Pellegrino ED, Veatch RM, Langan JP, eds. Ethics, Trust and the Professions: Philosophical and Cultural Aspects. Washington, DC: Georgetown University Press, 1991:45-63.

[12] Pellegrino ED, Thomasma DC. The Virtues in Medicine. New York: Oxford University Press, 1993.

[13] Sher G. What Makes a Lottery Fair? Noûs 1980;14 (2):203-16.

[14] Truog RD. Triage in the ICU. Hastings Cent Rep 1992;22:13-7. 1612881

[15] Brock DW. Health care resource prioritization and rationing: why is it so difficult? Social Research 2007;74(1):125-48.

[16] Truog RD. Screening mammography and the "R" word. N Engl J Med 2009;361:2501-3. 10.1056/NEJMp0911447 19940292

[17] Brock DW. A critique of three objections to physician-assisted suicide. Ethics 1999;109(3):519-47. 10.1086/233920 11657610

[18] Eyal N. Informed consent. In: Zalta EN, ed. Stanford Encyclopedia of Philosophy. 2011.

[19] Annas GJ. Standard of care--in sickness and in health and in emergencies. N Engl J Med 2010;362:2126-31. 10.1056/NEJMhle1002260 20519685

[20] Fink S. The deadly choices at Memorial. Published August 27, 2009

[21] Fink S. Doctors Face Ethical Decisions in Haiti. Published February 23, 2010.

[22] O'Reilly KB. Disaster medicine dilemmas examined. Published January 12, 2012.

[23] Williams B. A critique of utlitarianism. In: Williams B, Smart J, eds. Utilitarianism - for and against. 1st ed. Cambridge: Cambridge University Press, 1973:77-150.

[24] Scheffler S. Prerogatives without restrictions. Philosophical Perspectives 1992;6:377-97.

[25] Kelen GD, Kraus CK, McCarthy ML, et al. Inpatient disposition classification for the creation of hospital surge capacity: a multiphase study. Lancet 2006;368:1984-90. 10.1016/S0140-6736(06)69808-5 PMC7138047 17141705

[26] Merin O, Ash N, Levy G, Schwaber MJ, Kreiss Y. The Israeli Field Hospital in Haiti -- Ethical Dilemmas in Early Disaster Response. N Engl J Med 2010; 362:e38. 10.1056/NEJMp1001693 20200362

[27] Goodman A. Ministry of Touch -- Reflections on Disaster Work after the Haitian Earthquake. N Engl J Med 2010;362:e37. 10.1056/NEJMp1002311 20200361

[28] Fink S. India: Rationing in Disasters. In: Wikler D, Fink S, eds. Rationing health - who lives? who dies?: PRI The World, 2010:1-8.

[29] Firth PG. Standard of care--in sickness and in health and in emergencies. N Engl J Med 2010;363:1379-80; author reply 1380. 10.1056/NEJMc1007700 20886674

[30] Etienne M, Powell C, Amundson D. Healthcare ethics: the experience after the Haitian earthquake. Am J Disaster Med. Am J Disaster Med 2010;5(3):141-7. 20701171

[31] Smith RM, Dyer GS, Antonangeli K, et al. Disaster triage after the Haitian earthquake. Injury 2011;Aug 23. 10.1016/j.injury.2011.07.015 21868011

[32] Satterthwaite PS, Atkinson CJ. Using 'reverse triage' to create hospital surge capacity: Royal Darwin Hospital's response to the Ashmore Reef disaster. Emerg Med J 2012;29:160-2. 10.1136/emj.2010.098087 21030549

